# A Case Series of Rheumatoid Arthritis Flare Including Extra-articular Manifestations Following SARS-CoV-2 mRNA Vaccination: A Comprehensive Cytokine Assay

**DOI:** 10.7759/cureus.58740

**Published:** 2024-04-22

**Authors:** Narumichi Iwamura, Katusmi Eguchi, Ayuko Takatani, Kanako Tsutsumi, Tomohiro Koga, Takeshi Araki, Toshiyuki Aramaki, Kaoru Terada, Yukitaka Ueki

**Affiliations:** 1 Department of Internal Medicine, Sasebo Chuo Hospital, Sasebo, JPN; 2 Department of Rheumatology, Sasebo Chuo Hospital, Sasebo, JPN; 3 Department of Immunology and Rheumatology, Nagasaki University Graduate School of Biomedical Sciences, Nagasaki, JPN

**Keywords:** rheumatoid arthiritis, covid-19 vaccine, il-6 amplification, cytokine storm syndrome, cytokine assay, sars-cov-2 mrna vaccine, rheumatoid arthritis flare

## Abstract

Introduction: The administration of severe acute respiratory syndrome coronavirus 2 (SARS-CoV-2) mRNA vaccines has played a pivotal role in managing the COVID-19 pandemic. Nonetheless, there have been instances of atypical immune reactions to the vaccine, notably among patients with autoimmune inflammatory rheumatic diseases such as rheumatoid arthritis (RA).

Aim: This study was designed to analyze the cytokine profiles of RA patients who suffered from severe or fatal disease flares after receiving the SARS-CoV-2 mRNA vaccine, to unravel the immunological bases for such responses.

Methods: We conducted a retrospective observational study involving three RA patients. These individuals had their disease under control prior to experiencing severe disease flares post-mRNA vaccination. A detailed serum cytokine analysis was carried out and compared with that of a healthy control group.

Results: Post-vaccination, each patient displayed a marked cytokine storm, with notably increased levels of IL-1β (342, 109, and 27.5 pg/mL, respectively), IL-6 (67.8, 82.7, and 201 pg/mL, respectively), IL-17A (172, 51.6, and 30.3 pg/mL, respectively), and TNF-α (279, 97.5, and 59.4 pg/mL, respectively). Two patients responded well to treatment with biological and synthetic DMARDs, including baricitinib and abatacept. Unfortunately, one patient passed away even after receiving tocilizumab.

Conclusion: The findings from the comprehensive cytokine assays indicate severe cytokine abnormalities, pointing to cytokine storm syndrome. This suggests that SARS-CoV-2 mRNA vaccination may trigger a disruption in immune homeostasis, potentially leading to the acute worsening of pulmonary complications in RA patients, even those with previously low disease activity. It's necessary to weigh the risks of severe outcomes from COVID-19 against the potential for flares or other adverse reactions following vaccination. Such risk assessments should take into account the individual patient's health status, existing conditions, and other risk factors. Close follow-up after vaccination is crucial, especially for patients with RA.

## Introduction

Severe acute respiratory syndrome coronavirus 2 (SARS-CoV-2), a novel coronavirus that emerged in Wuhan, China, in December 2019, has led to the global spread of the infection known as COVID-19 [1]. Vaccination against SARS-CoV-2 has become a key strategy in preventing infection and severe outcomes of the disease. As of April 1, 2024, around 70.6% of the global population has received at least one dose of a COVID-19 vaccine, with approximately 13.57 billion doses administered worldwide and 8,948 doses being given each day [2]. Although vaccines have been crucial in combating the pandemic, there have been reports of abnormal immune responses in some instances. A national retrospective cohort study by Ma et al., involving 4,627 autoimmune inflammatory rheumatic disease patients, including 1,943 with rheumatoid arthritis (RA), found that 18% of patients experienced a flare following SARS-CoV-2 mRNA vaccination, with 14% requiring hospitalization or significant medication adjustments [3]. Terracina and Tan highlighted a case of a 55-year-old White male with non-erosive seropositive RA, who had been in sustained clinical remission for over two years but experienced an acute RA flare 12 hours after receiving his second BNT162b2 vaccine dose. Despite being in remission on upadacitinib monotherapy since July 2018, he developed significant symptoms after the vaccine that required medical attention [4]. Similarly, Bixio et al. conducted a prospective study of 77 RA patients in clinical remission, showing a 7.8% flare rate post-vaccination, mostly after the second dose, with the flares generally resolving within two weeks through standard treatments [5]. Younis et al. reported on the safety of various COVID-19 vaccines (Pfizer, Sinopharm, and AstraZeneca) among rheumatic disease patients in Iraq, finding flare rates of 9.9% to 10.3%, with no significant difference among vaccine types [6]. Additionally, we have previously described a case of a 61-year-old man without any autoimmune disease history who developed hypocomplementemic urticarial vasculitis and hemophagocytic lymphohistiocytosis (HLH) following the Moderna mRNA-1273 vaccine, suggesting that mRNA vaccines might trigger cytokine storm syndromes in susceptible individuals [7]. This paper aims to explore the hypothesis that mRNA vaccine-triggered cytokine abnormalities may play a role in severe or lethal RA flares, as evidenced by three well-controlled RA patients who experienced significant flares following SARS-CoV-2 mRNA vaccination, alongside comprehensive serum cytokine assay results.

After obtaining consent, serum cytokine samples from patients 1, 2, and 3 were obtained from our hospital. Serum cytokine levels were measured using the following steps. Serum samples were centrifuged at 3,000 × g for five min, after which the supernatants were collected and stored at -80°C for a maximum of 90 days before analysis. A blinded multiplex cytokine bead assay was performed in parallel using MILLIPLEX® MAP Human Cytokine/Chemokine Magnetic Bead Panel 1-Premixed 41 Plex (Millipore, Billerica, United States) kits, according to the manufacturer’s instructions. Cytokines that were frequently found to be at non-detectable levels were excluded from the analysis. A healthy group (n = 118) was recruited from staff at Nagasaki University and residents of the town of Saza in Nagasaki Prefecture, as previously described [8]. We presented the data on the following cytokines: IL-1 α, IL-1 β, IL-4, IL-6, IL-13, IL-17A, IL-17F, tumor necrosis factor (TNF)-α, interferon (IFN)-α2, IFN-γ, vascular endothelial growth factor (VEGF)-A, and granulocyte-macrophage colony-stimulating factor (GM-CSF).

## Case presentation

Case 1

An 80-year-old woman was diagnosed with RA based on the American College of Rheumatology (ACR)/European League Against Rheumatism (EULAR) classification criteria (2010) [9] on March, X-7 years. Rheumatoid factor (RF) at onset was positive, whereas anti-CCP antibody was negative. She was in remission for five years on subcutaneous tocilizumab (TCZ-SC) at 162 mg/2 weeks and tacrolimus at 2 mg/day.

**Figure 1:**
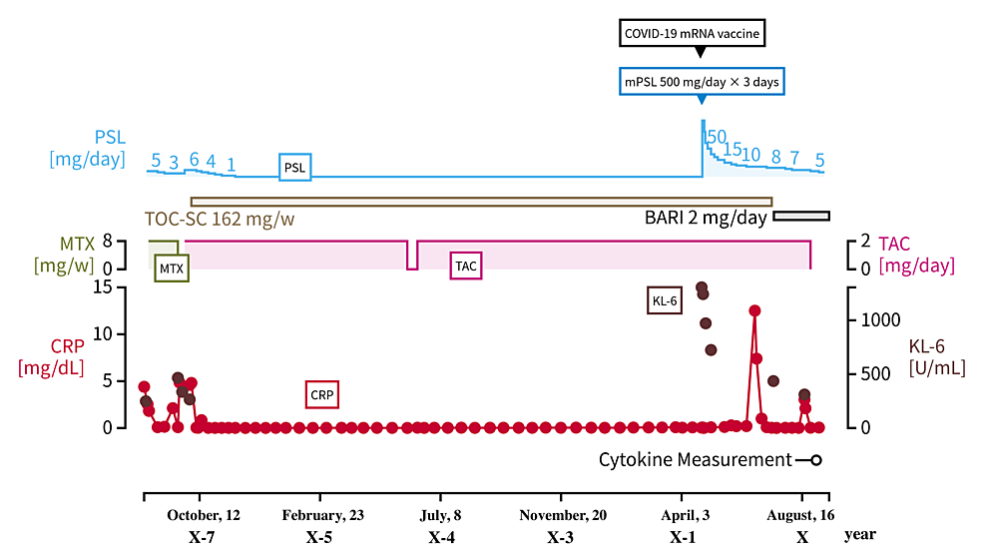
The clinical course and management of case 1 PSL: prednisolone; mPSL: methylprednisolone; TOC-SC: tocilizumab subcutaneous injection; BARI: baricitinib; MTX: methotrexate; TAC: tacrolimus; CRP: c-reactive protein; KL-6: sialylated carbohydrate antigen KL-6

Figure 1 presents her clinical course and treatment.

Her last computed tomography (CT) scan, taken on January 29, X-1 years before the vaccination, showed few abnormal findings, except faint fibrotic lesions just below the pleura in both lungs. In addition, during her last outpatient visit before the vaccination, she did not complain of any symptoms, including arthralgia or shortness of breathing, and her blood test showed no abnormalities, as evidenced by a c-reactive protein (CRP) level of 0.064 mg/dL on May 18, X years. The day after her first vaccination with the Pfizer-BioNTech vaccine (BNT-162b2) on July 20, X years, she experienced dyspnea requiring oxygen administration, and her chest CT showed new extensive reticular shadows and ground-glass opacity in both lungs. In addition, her blood test showed remarkably elevated sialylated carbohydrate antigen KL-6 (KL-6) levels (1306 U/mL) on July 25, X years compared with that at September, X-6 years (265 U/mL). RF (1984 IU/mL) was remarkably elevated, and hypocomplementemia was observed. Other blood test results are presented in Table 1.

**Table 1:** Disease activity of RA and laboratory data before and after vaccination DAS28-ESR: disease activity score erythrocyte sedimentation rate; DAS28-CRP: disease activity score 28-c-reactive protein; CDAI: clinical disease activity index; SDAI: simplified disease activity index; RA: rheumatoid arthritis

	Unit	Reference range	Case 1		Case 2		Case 3	
			Before first vaccination	After administration of methylprednisolone	Before second vaccination	After second vaccination	Before third vaccination	After third vaccination
			May 18, X	June 28, X	March 12, Y-1	April 1, Y	April 12, Z	September 5, Z
			33 days before	8 days after	375 days before	10 days after	127 days before	19 days after
Disease activity of RA								
DAS28-ESR		<2.6	2.76	2.01 (July 7, X)	2.51	5.45	3.65	N/A
DAS28-CRP		<2.3	2.11	1.08 (July 7, X)	1.84	5.06	3.17	N/A
CDAI		≦2.8	4.90	1.00 (July 7, X)	1.50	25.1	10.7	N/A
SDAI		≦3.3	4.80	1.00 (July 7, X)	0.600	20.0	10.5	N/A
Laboratory data								
White blood cell	/μL	3.30-8.60	5600	19000	4300	5500	6900	7800
Red blood cell	×10^6^/μL	3.77-5.55	4.05	3.60	4.04	4.06	3.80	4.07
Hemoglobin	g/dl	13.7-16.8	12.7	11.5	12.1	11.4	12.5	12.9
Platelet	×10^4^/μL	15.8-34.8	15.8	15.1	22.7	30.3	19.4	22.2
Neutrophil	%	38.0-58.9	58	92	53	66	64.8	66
Eosinophil	%	0.00-6.80	4.9	0.0	8.0	3.2	1.3	2.6
Basocyte	%	0.00-2.00	0.80	0.10	0.60	0.50	0.8	1.1
Lymphocyte	%	26.0-46.6	26	5.6	23	20	23.3	14
Monocyte	%	2.30-7.70	11	2.1	15	11	9.8	17
Erythrocyte sedimentation rate	mm/1h	<15	13	19	34	53	14	N/A
Total protein	g/dL	6.6-8.1	6.3	5.5	6.7	7.0	7.2	7.2
Albumin	g/dL	4.1-5.1	3.8	3.1	3.6	3.2	4.0	3.5
Sodium	mmol/L	138-145	140	138	141	144	141	134
Potassium	mmol/L	3.6-4.8	4.4	4.4	4.3	4.1	3.6	3.3
Chlorine	mmol/L	101-108	105	108	107	110	104	97
Urea nitrogen	mg/dL	8.0-20	17	23	23	20	16	12
Creatinine	mg/dL	0.65-1.1	0.91	1.0	0.95	0.92	0.65	0.60
Aspartate aminotransferase	U/L	13-30	20	17	20	17	60	31
Alanine aminotransferase	U/L	10-42	17	13	11	9.0	47	21
Lactate dehydrogenase	U/L	124-222	165	281	170	155	267	275
Total-bilirubin	mg/dL	6.6-8.1	0.40	0.30	0.70	0.60	0.60	1.2
Alkaline phosphatase	U/L	38-113	79	52	N/A	68	N/A	84
γ-glutamyl transpeptidase	U/L	13-64	11	10	16	14	24	28
C-reactive protein	mg/dL	0.00-0.14	0.064	0.063	0.92	5.1	0.21	5.9
Complement component 3	mg/dL	73-138	N/A	67	N/A	100	N/A	N/A
Complement component 4	mg/dL	11-31	N/A	7.4	N/A	26	N/A	N/A
50% hemolytic unit of complement	U/mL	32-58	N/A	<10	N/A	53	N/A	N/A
Rheumatoid factor	IU/ml	<15	365 (September 1, X-6)	1980 (July 28, X)	153 (September 14, Y-4)	136	103 (July 31, Z-15)	54.8 (September 12, Z)

The Grace scale synovitis score indicated grade 2 synovitis at the proximal interphalangeal (PIP) joint of the third digit on the right hand. Grade 1 synovitis was observed at the metacarpophalangeal (MCP) joint and the interphalangeal (IP) joint of the first digit, the MCP joint of the second digit, the MCP joint of the third digit, the MCP joint of the fifth digit, and the wrist, whereas grade 0 was recorded for the remaining joints. On the left hand, the MCP joint of the third digit exhibited grade 2 synovitis, as did the MCP joints of the second and fourth digits, with grade 0 noted for the other joints. Fluid accumulation was present around the flexor tendons of both wrists and hands and around the extensor tendons of the right foot.

We have ruled out bacterial pneumonia and viral pneumonia, including pneumocystis, SARS-CoV-2, and cytomegalovirus pneumonia. A diagnosis of acute exacerbation of interstitial pneumonia caused by RA with vasculitis was then established based on the Japanese Ministry of Health, Labour, and Welfare diagnosis criteria [10].

We initiated the following treatment: methylprednisolone (mPSL) at 500 mg/day for three days, followed by prednisolone (PSL) at 50 mg/day, in addition to TCZ at 162 mg/2 weeks and tacrolimus at 2 mg/day on July 25, X years, five days after the vaccination. The ground-glass opacity in both her lungs tended to shrink, and her dyspnea requiring oxygen administration resolved. However, after reducing her PSL dosage to 8 mg/day, she experienced an exacerbation of joint pain in the extremities without any other incitements, such as infection, on February 1, X + 1 year, seven months after vaccination. PSL at 9 mg/day was then administered in addition to TCZ at 162 mg/2 weeks and tacrolimus at 2 mg/day, after which her blood test results showed a remarkable elevation in CRP levels (12.5 mg/dL). As such, a relapse of RA was diagnosed, for which baricitinib at 2 mg/day instead of TCZ was administered on April 21, X + 1 year, nine months after initiating mPSL treatment.

Following the administration of baricitinib at 2 mg/day in addition to PSL at 7 mg/day and tacrolimus 2 at mg/day for the treatment of poorly controlled RA, her complaints of joint pain ceased. Her blood test showed a decrease in both CRP (3.04 mg/dL) and KL-6 (311 U/mL) levels on August 26, X + 1 year, four months after initiating baricitinib and PSL treatment. Cytokine measurements on October 14, X + 1 year, 17 months after her vaccination, showed the following results: IL-1β, 342 pg/mL; IL-6, 67.8 pg/mL; IL-17A, 172 pg/mL; and TNF-α, 279 pg/mL (Table 2).

**Table 2:** Results of the comprehensive cytokine assay *: abnormal values exceeding the third quartile of the control group; †: abnormal values exceeding the median + 2SD (standard deviation) of the control group; IL‡: interleukin; TNF§: tumor necrosis factor; IFN||: interferon; VEGF¶: vascular endothelial growth factor; GM-CSF⁑: granulocyte-macrophage colony-stimulating factor

	Unit	Median (first quartile, third quartile) of control group (n = 118)	Case 1	Case 2	Case 3
Time from last vaccination to sample collection (weeks)			68	8	3
Age	years	56.0 (47.0-65.5)	80	78	75
IL^‡^-1α	pg/mL	10.2 (0.00-8.70)	480^†^	104^*^	46.8^*^
IL^‡^-1β	pg/mL	0.00 (0.00-1.15)	342^†^	109^†^	27.5^†^
IL^‡^-4	pg/mL	0.00 (0.00-6.62)	39.5^*^	11.7^*^	11.4^*^
IL^‡^-6	pg/mL	0.00 (0.00-0.00)	67.8^†^	82.7^†^	201^†^
IL^‡^-13	pg/mL	0.00 (0.00-0.00)	609^†^	333^*^	123^*^
IL^‡^-17A	pg/mL	9.11 (0.260-6.15)	172^†^	51.6^†^	30.3
IL^‡^-17F	pg/mL	0.00 (0.260-6.15)	233^†^	36.4^*^	246^†^
TNF^§^-α	pg/mL	11.2 (9.27-16.6)	279^†^	97.5^†^	59.4^†^
IFN^||^-α2	pg/mL	0.00 (0.00-0.00)	849^†^	287^†^	96.9^*^
IFN^||^-γ	pg/mL	4.74 (1.50-11.3)	129^†^	21.7^*^	62.9^†^
VEGF^¶^-A	pg/mL	99.4 (31.9-209)	319^*^	259^*^	337^*^
GM-CSF^⁑^	pg/mL	1.52 (0.00-6.70)	98.8^†^	27.8^*^	18.8^*^

Case 2

**Figure 2:**
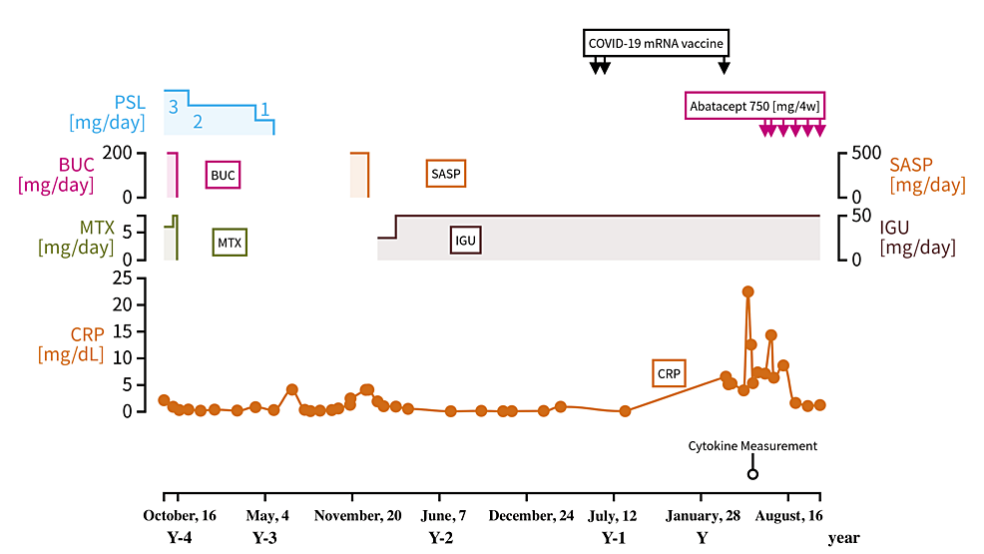
The clinical course and management of case 2 PSL: prednisolone; BUC: bucillamine; SASP: salazosulfapyridine; MTX: methotrexate; IGU: iguratimod; CRP: c-reactive protein

A 78-year-old man was diagnosed with RA based on the ACR/EULAR classification criteria (2010) [9] in Y-17 years. RF at onset was 1441 IU/mL, while anti-CCP antibodies were negative. He had been treated for RA with medications including Bucillamine 200 mg/day, Methotrexate 5-6 mg/day, and Prednisolone 0-3 mg/day until October of year X-4. However, since February 28, X-2 year, he has been maintaining low disease activity with only Iguratimod 50 mg/day. Figure 2 displays his time course and treatment.

He received the first and second Moderna vaccinations (mRNA-1273) on May 31, Y-1 years, and June 21, Y-1 years, respectively. He did not complain of any significant symptoms following these vaccinations, and both his blood test and chest X-ray showed no significant changes, as evidenced by a CRP level of 0.109 mg/dL on August 7, Y years, six weeks after the second vaccination. However, 10 days after the third vaccination with the Moderna mRNA vaccine (mRNA-1273) on March 22, Y years, he developed systemic arthralgia and marked pleural effusion on chest X-ray. He was referred to our hospital with suspicion of an RA relapse. His blood test showed elevated CRP levels (5.13 mg/dL) 10 days after his last vaccination. His CRP levels increased even higher eight weeks after his last vaccination (22.4 mg/dL). Other blood test results are presented in Table 1. At the time of visit five days after vaccination, the RF and matrix metalloproteinase-3 values were 146 IU/mL and 160 ng/mL, respectively. Magnetic resonance image of the wrist revealed a T1 low signal and short-TI inversion recovery high signal suggesting bone marrow edema, in the distal ends of the radius and ulna to the carpals and proximal metacarpals of both hands. Furthermore, edema of the subcutaneous lipid tissue was prominent, mainly on the dorsal surfaces of the hands. The simplified disease activity index (SDAI) increased from 1.5 to 25.1, and the clinical disease activity index (CDAI) increased from 0.6 to 20 when compared with the values obtained one year ago. The erythrocyte sedimentation rate increased from 34 to 53 mm/h and CRP from 1.84 to 5.13 mg/dL compared with 375 days before vaccination.

Infectious diseases such as bacterial pleurisy and tuberculous pleurisy, malignant diseases such as cancerous pleurisy and malignant pleural mesothelioma, and collagen diseases such as RA are the differential diagnoses of exudative pleural effusion. We performed thoracentesis on the pleural effusion, which appeared yellow and serous; the cell count was below the threshold. Both adenosin deaminase and antimicrobial smears yielded negative results; pleural fluid culture did not detect any bacteria; and cytology results were negative. We considered infection or malignancy could be ruled out as the causes of exudative pleural effusion. Given the history of increased pleural effusion with worsening RA, we concluded that the exudative effusion was most likely rheumatoid pleural effusion.

We introduced an intravenous infusion of abatacept 750 mg/2 weeks on June 10, Y years, following which his symptoms of systemic arthralgia and pleural effusion were reduced. Cytokine measurements on May 27, Y years, eight weeks after his third vaccination, showed the following results: IL-1β, 109 pg/mL; IL-6, 82.7 pg/mL; IL-17A, 51.6 pg/mL; and TNF-α, 97.5 pg/mL (Table 2).

Case 3

**Figure 3:**
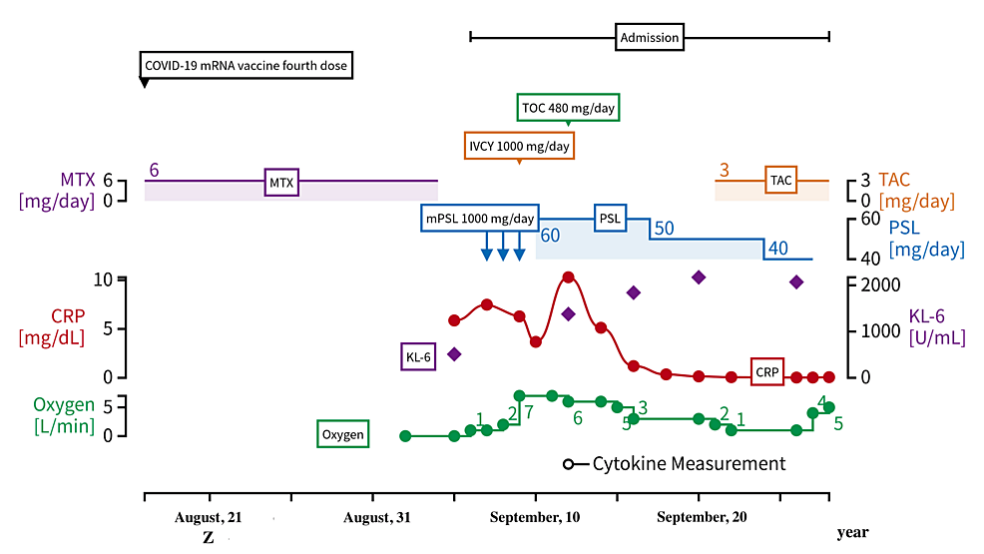
The clinical course and management of case 3 TOC: tocilizumab; IVCY: intravenous cyclophosphamide; MTY: methotrexate; TAC: tacrolimus; mPSL: methylprednisolone; PSL: prednisolone; CRP: c-reactive protein; KL-6: sialylated carbohydrate antigen KL-6

A 75-year-old woman was administered methotrexate (MTX) at 6 mg/week for RA diagnosed based on the ACR/EULAR classification criteria (2010) [9] in Z-15 years. Figure 3 displays her time course and treatment.

Since then, she has maintained low disease activity under MTX administration. She received two doses of the Pfizer-BioNTech mRNA vaccine (BNT162b2) in Z-1 years and did not experience any significant symptoms after vaccination. However, following her third vaccination with the Moderna mRNA vaccine (mRNA-1273) in late May (Z years), she developed intense fatigue and mild shortness of breath, which lasted for a week. Despite her symptoms, she did not seek medical attention. Moreover, she received the fourth dose of the Moderna mRNA vaccine (mRNA-1273) on August 17, Z years. The day after her vaccination, she developed fever, shortness of breath during physical activity, and intense fatigue, which worsened over time. In addition, three days after her vaccination, she complained of coughing, headache, and postprandial vomiting. These symptoms worsened over time, resulting in dyspnea even at rest. At this stage, she decided to visit us and was admitted to our hospital on September 7, Z years, three weeks after her last vaccination.

Chest CT showed diffuse heterogeneous ground-glass opacity in both lungs, which was suspicious for a DAD pattern. Her blood test showed elevated CRP levels (5.86 mg/dL) on September 5, Z years. No significant decrease in serum complement titers was observed. The other results of her blood test are presented in Table 1. The bronchoalveolar lavage fluid (BALF) findings were averaged from fluid 1 to fluid 3, with a cell count of 53 × 104/ml, of which 4.9% were macrophages, 71.0% were lymphocytes, 16.7% were neutrophils, and 6.9% were eosinophils. The SARS-CoV-2 PCR test using BALF was negative, as was the antimicrobial smear.

We considered pneumocystis pneumonia (PCP), MTX pneumonia, bacterial pneumonia, cytomegalovirus pneumonia, SARS-CoV-2 pneumonia, serum sickness, and rheumatoid arthritis-associated interstitial lung disease (RA-ILD) as differential diagnoses. We ruled out PCP because blood tests on admission showed negative β-D glucan. We considered bacterial pneumonia unlikely, as blood tests upon admission showed no significant elevation of white blood cells and procalcitonin. However, we could not completely rule out the possibility of bacterial pneumonia; therefore, ampicillin sodium 3 g/day and levofloxacin 500 mg/day were administered, with no obvious changes in the chest X-ray findings. Viral pneumonia, including SARS-CoV-2 and cytomegalovirus, was also ruled out based on the negative viral test results. Based on the course of fever and polyarthralgia that developed following vaccination, we considered serum sickness as a differential disease, although it is a rare condition. However, serum sickness seemed unlikely due to the absence of decreased serum complement titers and the nontypical appearance of pulmonary lesions. Although it is typical that the administration of steroids can induce rapid and complete remission in patients with serum sickness [11], lung lesions did not improve after three days of mPSL 1000 mg/day administration in this case; thus, serum sickness was ruled out. We considered the interstitial pneumonia to have been caused by RA-ILD, which was triggered by the SARS-CoV-2 mRNA vaccine. Although pulmonary involvement in RA is usually in the form of the usual interstitial pattern, we speculated that severe inflammation caused the acute presentation of pulmonary involvement in this case. RA-ILD often presents with acute interstitial pneumonia with organizing pneumonia and a diffuse alveolar damage (DAD) pattern. On CT, the interstitial pneumonia in this case showed a DAD pattern, which was predicted to be resistant to immunosuppressive therapy.

We introduced mPSL at 1000 mg/day for three days, followed by PSL at 60 mg/day for RA-ILD 21 days after her vaccination. Owing to the poor response to corticosteroids, we introduced cyclophosphamide at 1000 mg/day for a day. Despite these strong immunosuppressive therapies, the patient’s condition deteriorated over time, as evidenced by an elevation of CRP levels from 3.68 to 10.3 mg/dL, an elevation of KL-6 levels from 510 to 1378 U/mL, and an increased need for oxygen from 1 to 7 L/min six days after admission. After determining that the aforementioned immunosuppressive therapies were ineffective, we administered TCZ (480 mg/day) 26 days after her vaccination based on noticeably elevated CRP levels, which suggested that IL-6 was involved in immune abnormalities in this patient. The patient’s dyspnea improved over time, and the requirement for oxygen decreased to 1 L/min after TCZ administration. As a matter of course, CRP levels decreased to 0.059 mg/dL. Furthermore, interstitial pneumonia with a DAD pattern tended to disappear 10 days after TCZ administration. Unfortunately, the patient died of asphyxiation due to aspiration of vomit during SARS-CoV-2 PCR testing. Cytokine measurements 3 weeks after the fourth vaccination showed the following results: IL-1β, 27.5 pg/mL; IL-6, 201 pg/mL; IL-17A, 30.3 pg/mL; and TNF-α, 59.4 pg/mL (Table 2).

## Discussion

In this study, we experienced three cases of RA flares including extra-articular symptoms following SARS-CoV-2 mRNA vaccination. There have been previous reports of RA flares following vaccination [3-7]. The treatment for RA was not interrupted at all during the time of vaccination in those three cases. It may be possible that SARS-CoV-2 mRNA vaccinations were involved with RA flares in the three cases we experienced in this study. Ma et al. conducted a national retrospective cohort study of consecutive autoimmune inflammatory rheumatic disease patients who received at least one dose of a COVID-19 mRNA vaccine, targeting 4627 patients including 1943 patients with RA [3]. According to this study, 18% of patients flared after SARS-CoV-2 mRNA vaccination, of which 14% required hospitalization or glucocorticoids more than prednisolone equivalent of 20 mg/day, or new initiation of a biological or cytotoxic agent. All three cases of post-vaccination RA flares we describe required hospitalization for more than 14 days and the introduction of new biological disease-modifying anti-rheumatic drugs (bio-DMARDs), one of which resulted in fatal. The results of the above study show that patients with inflammatory arthritis had a higher risk of flare (HR 1.5 (1.2-2.0)) [3]. The aforementioned study also showed that treatment with conventional synthetic disease-modifying anti-rheumatic drugs (csDMARDs), immunosuppression, and prednisolone was also associated with an increased risk of flare (HR 1.5 (1.1-2), 1.2 (1.1-1.4) and 1.5 (1.2-1.8) for prednisolone ≤7.5 mg, respectively) [3]. Prior to the mRNA vaccination, case 1 had received TCZ-SC and tacrolimus, case 2 had received iguratimod, and case 3 had received MTX to control RA. One of the factors that may have contributed to RA flare in our experience was the underlying RA that required the administration of conventional synthetic or bio-DMARDs.

Patients with RA are frequently treated with immunosuppressants and biologics and may therefore be at risk of COVID-19 [12]. Glucocorticoids and combination therapy with immunomodulators and biologics increase the risk of severe COVID-19 outcomes [13]. Therefore, SARS-CoV-2 mRNA vaccination has been more strongly recommended in patients with RA than in the general population. The European Alliance of Associations for Rheumatology stated that they see no reason why patients with rheumatic diseases should be excluded from vaccination [14]. The ACR strongly recommends vaccination [15]. Japanese College of Rheumatology also stated that vaccination of patients with collagen and rheumatic diseases, including additional (third and fourth) doses, is worthy of careful consideration [16].

**Figure 4:**
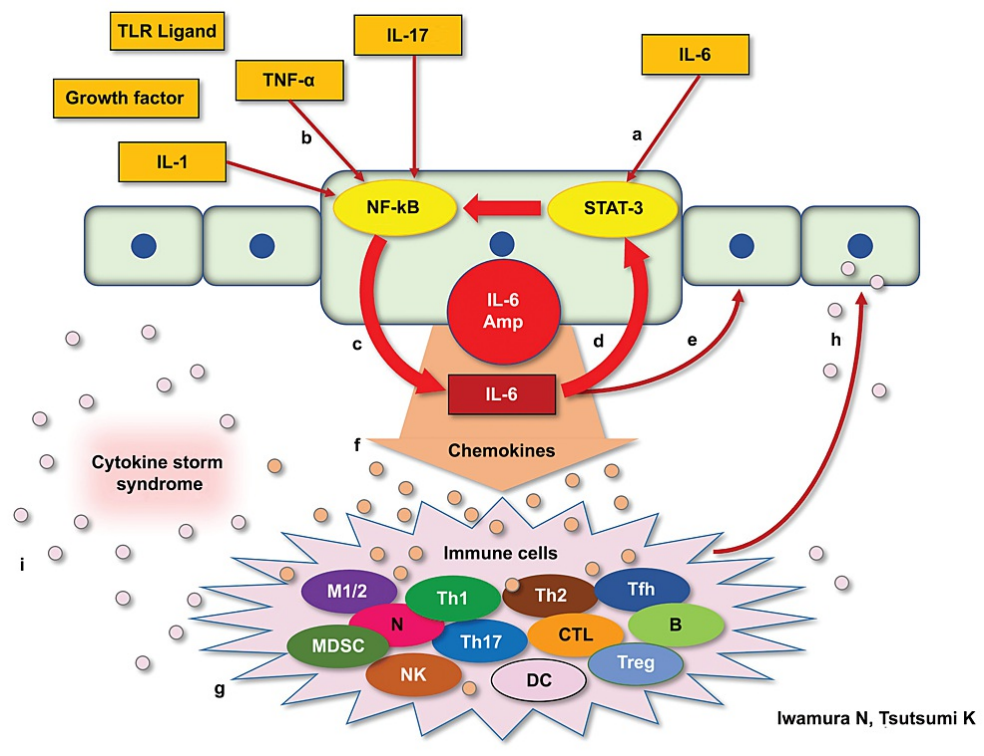
IL-6 Amp mechanism IL-6 stimulates an intracellular signaling pathway: STAT-3 (A), whereas TNF-α, IL-17, IL-1, TLR ligands, and growth factors stimulate another intracellular signaling pathway: NF-κB (B). In nonimmunocompetent cells, when STAT-3 and NF-κB are stimulated at the same time, NF-κB becomes excessively activated, resulting in excessive production of IL-6 (C). Large numbers of IL-6 act via autocrine (D) and paracrine (E) signaling, promoting much more IL-6 production following STAT-3 activation. We defined these positive feedback mechanisms that mediate IL-6 as “IL-6 Amp” in a narrow sense. Nonimmunocompetent cells activated by IL-6 Amp produce various types of chemokines (F). These chemokines stimulate immunocompetent cells such as macrophages, T cells, and B cells (G). Various types of chemokines produced by these immunocompetent cells stimulate STAT-3 and NF-κB in nonimmunocompetent cells again (H), which consequently produce much more IL-6 and chemokines. We defined this mechanism as “IL-6 Amp” in a broad sense. Thus, IL-6 Amp can occur when adequate amounts of both IL-6 to activate STAT-3 and TNF-α and IL-1 or IL-17 to activate NF-κB are present. Throughout the course of IL-6 Amp, tremendous numbers of cytokines and chemokines are produced, which we call “cytokine storm syndrome” (I). TNF: tumor necrosis factor; STAT-3: signal transducer and activator of transcription 3; TLR: toll-like-receptors; MDSC: myeloid-derived suppressor cell; DC: dendritic cell; TCL: tumor cell lysate

All three cases showed abnormal values of IL-1α and IL-1β. In addition, IL-6 and TNF-α values measured in all three cases were above the median and double the SD of those in the healthy group. IL-17 values were above the third quartile of those in the healthy group. Case 1 and case 3 showed acute exacerbation of interstitial pneumonia, and case 2 and case 3 showed elevated CRP levels; inflammatory cytokines, including IL-6, were elevated in case 1 and case 2, even after therapeutic intervention. These results supported the presence of a cytokine storm in all cases. Based on the cytokine profiles of these cases, we speculated that IL-6 Amp might be one of the mechanisms by which mRNA vaccination was involved with cytokine storm in these cases [17]. IL-6 Amp is a mechanism by which simultaneous activation of NF-κB and STAT3 in non-immune cells triggers a positive feedback loop of NF-κB activation through the IL-6-STAT3 axis. We demonstrate the detailed mechanism of the IL-6 Amp in Figure 4 [7].

The cytokine assay results of these cases indicate that IL-6 Amp can occur when there is enough IL-6 to activate STAT-3 and TNF-α and IL-1 or IL-17 to activate NF-κB.

Among the three cases of RA flare following the SARS-CoV-2 mRNA vaccination presented here, the introduction of baricitinib in case 1 and abatacept in case 2 resulted in the suppression of RA activity (Table 3).

**Table 3:** Cases of RA flares following SARS-CoV-2 mRNA vaccination F^*^: female; M^†^: Male; RA^‡^: rheumatoid arthritis; TCZ-SC^§^: subcutaneous tocilizumab; TAC^||^: tacrolimus; IGU^¶^: iguratimod; MTX^⁑^: methotrexate; IP^††^: interstitial pneumonia; BARI^‡‡^: baricitinib; ABT^§§^: abatacept; TCZ-IV^||||^: tocilizumab intravenous injection

Item	Unit	Case 1	Case 2	Case 3
Age		80	78	75
Sex	Years	F^*^	M^†^	F^*^
Underlying disease		RA^‡^	RA^‡^	RA^‡^
Years from the diagnosis of RA	Years	8	17	16
Years of having low disease activity	Years	5	2	11
Ongoing treatment for RA before admission		TCZ-SC^§ ^162 mg/2w + TAC^||^ 2 mg/day	IGU^¶^ 50 mg/day	MTX^⁑^ 6 mg/w
Number of vaccinations before admission	Times	1	3	3
Vaccine type		BNT-162b2 (Pfizer/BioNTech)	mRNA-1273 (Moderna)	mRNA-1273 (Moderna)
Days from the last vaccine dose before admission	Days	1	10	1
The type of flare		Acute exacerbation of IP^††^	Pleural effusion and arthritis	Acute exacerbation of IP^††^
Peak CRP value	mg/dL	12.5	22.5	10.3
Peak IL-6 levels	pg/mL	67.8	82.7	1420
Newly initiated DMARDs		BARI^‡‡^ 2 mg/day	ABT^§§^ 750 mg/4w	TCZ-IV^||||^ 480 mg/4w
Outcome		21 days of hospitalization	14 days of hospitalization	Death after 23 days of hospitalization

Although case 3 eventually died from aspiration of vomit, the introduction of tocilizumab resulted in a significant improvement in clinical symptoms and a significant reduction in oxygen requirement. No reports have examined the drug of choice for RA flares induced by SARS-CoV-2 mRNA vaccines. As can be seen from the cytokine measurements, despite some variation in the cytokine profile, our patients are thought to have experienced a severe cytokine storm through IL-6 Amp and other mechanisms. Therefore, a possible strategy is to administer tocilizumab to target IL-6 with the aim of reducing the amplification of cytokine storms by IL-6 Amp in the early stages of the cytokine storm. Other strategies may also involve the introduction of Janus kinase inhibitors, such as baricitinib or cytotoxic T-lymphocyte associated antigen 4 inhibitors, for cases with a highly advanced cytokine storm resulting in substantially increased production of various cytokines. However, given the difficulty of performing comprehensive cytokine measurements in general practice, for RA flares induced by SARS-CoV-2 mRNA vaccines, it seems reasonable to add one bio/cs DMARDs based on RA activity, drug use history, and experience of drug use in the institution until more evidence is accumulated.

Based on the time series, it may be possible that SARS-CoV-2 mRNA vaccination triggered RA flares in all three cases; however, the causal relationship between vaccination and RA flares cannot be proven. It is also a well-known fact that patients with RA present with immune abnormalities, which makes it impossible to determine whether the highly advanced cytokine abnormalities presented in Table 2 are caused by SARS-CoV-2 mRNA vaccines or by the immune abnormality associated with RA itself. This study also has a limitation in that the methodology is not rigorous enough; serum samples were collected at different time frames. To overcome this problem, cytokines need to be measured and compared before and after vaccination. In addition, we could not suggest a suitable treatment for RA flare following SARS-CoV-2 mRNA vaccination because the three cases presented here were treated differently; further studies are required. This is only a three-case report, so the evidence for the suggestions mentioned here is insufficient.

## Conclusions

We encountered three cases of severe or lethal RA flare that developed following the SARS-CoV-2 mRNA vaccination in RA patients with maintained low disease activity. Comprehensive cytokine assay results showed highly advanced cytokine abnormalities, whereas serological studies suggested the presence of cytokine storm syndrome. The introduction of baricitinib in case 1 and abatacept in case 2 suppressed RA activity. Although case 3 eventually died, the introduction of tocilizumab temporarily promoted a significant improvement in clinical symptoms and imaging findings.
